# Conservative management of nivolumab-induced pericardial effusion: a case report and review of literature

**DOI:** 10.1186/s40164-018-0104-y

**Published:** 2018-05-08

**Authors:** Shagufta Shaheen, Hamid Mirshahidi, Gayathri Nagaraj, Chung-Tsen Hsueh

**Affiliations:** Division of Hematology and Oncology, Department of Medicine, Loma Linda University, 11175 Campus Street, CSP 11015, Loma Linda, CA 92354 USA

**Keywords:** Non-small cell lung cancer, Immune checkpoint inhibitor, Nivolumab, Pericardial effusion, Corticosteroids

## Abstract

**Background:**

Nivolumab is an immune checkpoint inhibitor targeting programmed death-1 protein and has been approved for the treatment of multiple advanced malignancies. Adverse effects of immune checkpoint inhibitors are distinct from conventional cytotoxic chemotherapy and can be life-threatening if left unrecognized. Here, we present a case of nivolumab-induced pericardial effusion successfully managed with high-dose corticosteroids.

**Case presentation:**

A 70-year-old Caucasian female with a history of 50-pack-year cigarette smoking was diagnosed of recurrent adenocarcinoma of lung after initial surgery. She progressed through multiple lines of chemotherapy and was eventually started on nivolumab. She developed a large pericardial effusion, grade 3 by Common Terminology Criteria for Adverse Events v4.0, about 4 days after receiving first nivolumab treatment. She was treated with oral prednisone at 1 mg/kg daily with gradual resolution of pericardial effusion over 5 weeks while she still received nivolumab every 2 weeks. Prednisone treatment was eventually tapered off about 10 weeks from initial nivolumab treatment. However 1 week after stopping prednisone, she again presented with shortness of breath and bilateral ankle edema, imaging confirmed recurrent pericardial effusion measuring 2.8 cm. Nivolumab was stopped and patient was again started back on prednisone 1 mg/kg daily which resulted in complete resolution of pericardial effusion in 3 weeks. Nivolumab was resumed 1 week afterwards while patient was on tapering dose of prednisone. There was no recurrent pericardial effusion when she continued low-dose prednisone during the remaining course of nivolumab treatment.

**Conclusions:**

With increasing use of immune checkpoint inhibitors, clinicians need to be aware of the unusual immune-related adverse events in order to provide timely management and effective patient care. To our knowledge, this is the first reported case of immune-related pericardial effusion from nivolumab successfully managed with high-dose corticosteroids. Furthermore, recurrent pericardial effusion was prevented by using low-dose corticosteroids as maintenance in order for patient to continue nivolumab treatment.

## Background

Immune checkpoint inhibitors, including monoclonal antibodies against programmed death-1 (PD-1), ligands of PD-1 and cytotoxic T-lymphocyte associated antigen-4 (CTLA-4) have been approved in the treatment of advanced malignancies. PD-1 and CTLA-4 belong to superfamily of CD28. PD-1 delivers negative signals to T cells upon interaction with one of its two ligands, programmed death ligand 1 (PD-L1) or programmed death ligand 2 [1]. Binding of the PD-1 to its ligands inhibits kinases that are involved in T cell activation, and enable tumor cells to evade immune detection and destruction [2]. Nivolumab is an IgG4 antibody that targets PD-1 on the surface of T cells. Nivolumab blocks the interaction between PD-1 to its ligands, and allows T cells to recognize tumor cells and destroy them. Nivolumab has been approved by US FDA in the treatment of advanced malignancies such as melanoma, non-small cell lung cancer (NSCLC), renal cell carcinoma, urothelial cancer, squamous cell carcinoma of head and neck, and Hodgkin’s disease. Adverse events associated with nivolumab and other immune checkpoint inhibitors are different from cytotoxic chemotherapy. Since immune checkpoint inhibitors activate T cells, their adverse effects are mostly immune mediated reactions such as colitis, hepatitis, thyroiditis, hypophysitis, pneumonitis, pericarditis, skin rash etc. Here we report a case of pericardial effusion associated with nivolumab treatment in patient with metastatic NSCLC.

## Case presentation

A 70-year old female with cigarette smoking history of 50 pack years, presented with chronic cough and weight loss. CT scan of chest demonstrated a 4.7-cm right lower lobe lung mass. She was subsequently diagnosed with Stage Ib adenocarcinoma of lung and received right lung resection. Eighteen months later she developed recurrent disease in the lung and was treated with concurrent chemoradiation. Afterwards, she progressed with metastatic subcarinal, mediastinal and hilar lymphadenopathy. PET-CT and MRI of brain did not reveal extra-thoracic metastasis. Patient underwent biopsy of the mediastinal mass, which confirmed metastatic adenocarcinoma. There was no mutation in epidermal growth factor receptor gene, or translocation in anaplastic lymphoma kinase gene in her tumor. She progressed through several lines of treatment including paclitaxel plus carboplatin then vinorelbine alone. Thereafter she was referred to medical oncology clinic of our cancer center, and nivolumab treatment was recommended.

**Fig. 1:**
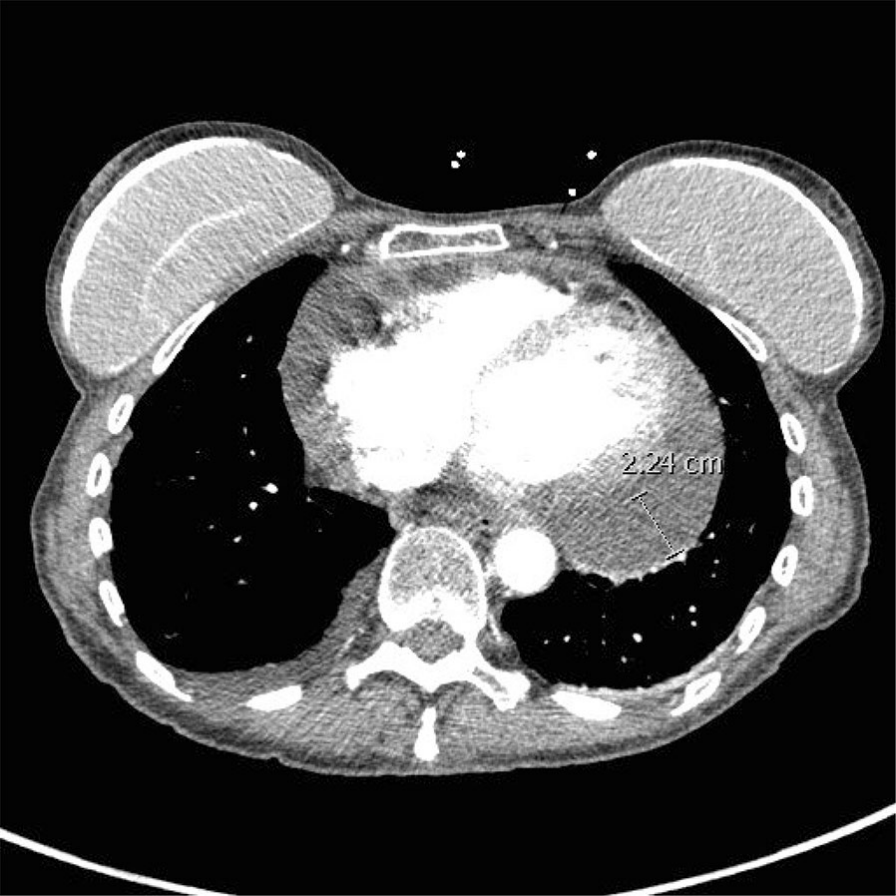
CT angiography of the chest demonstrated a large pericardial effusion of 2.4 cm thickness after treatment with first dose of nivolumab

**Fig. 2:**
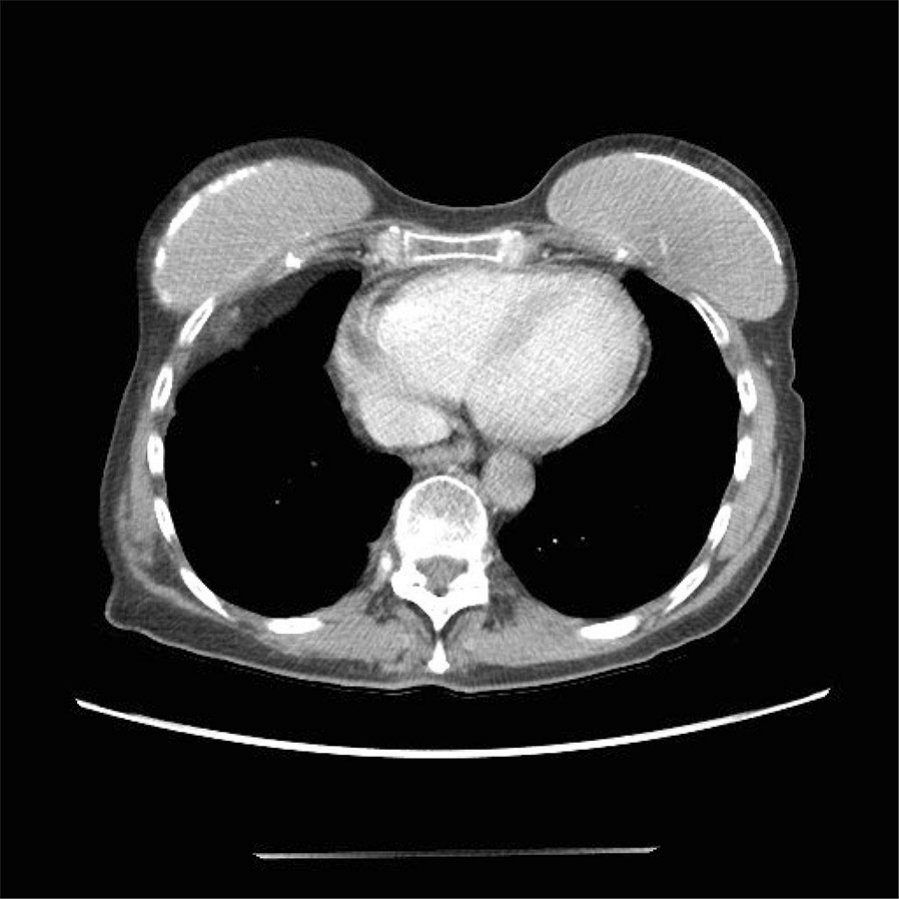
CT of the chest demonstrated complete resolution of pericardial effusion after 4 weeks of high-dose steroid treatment

**Fig. 3:**
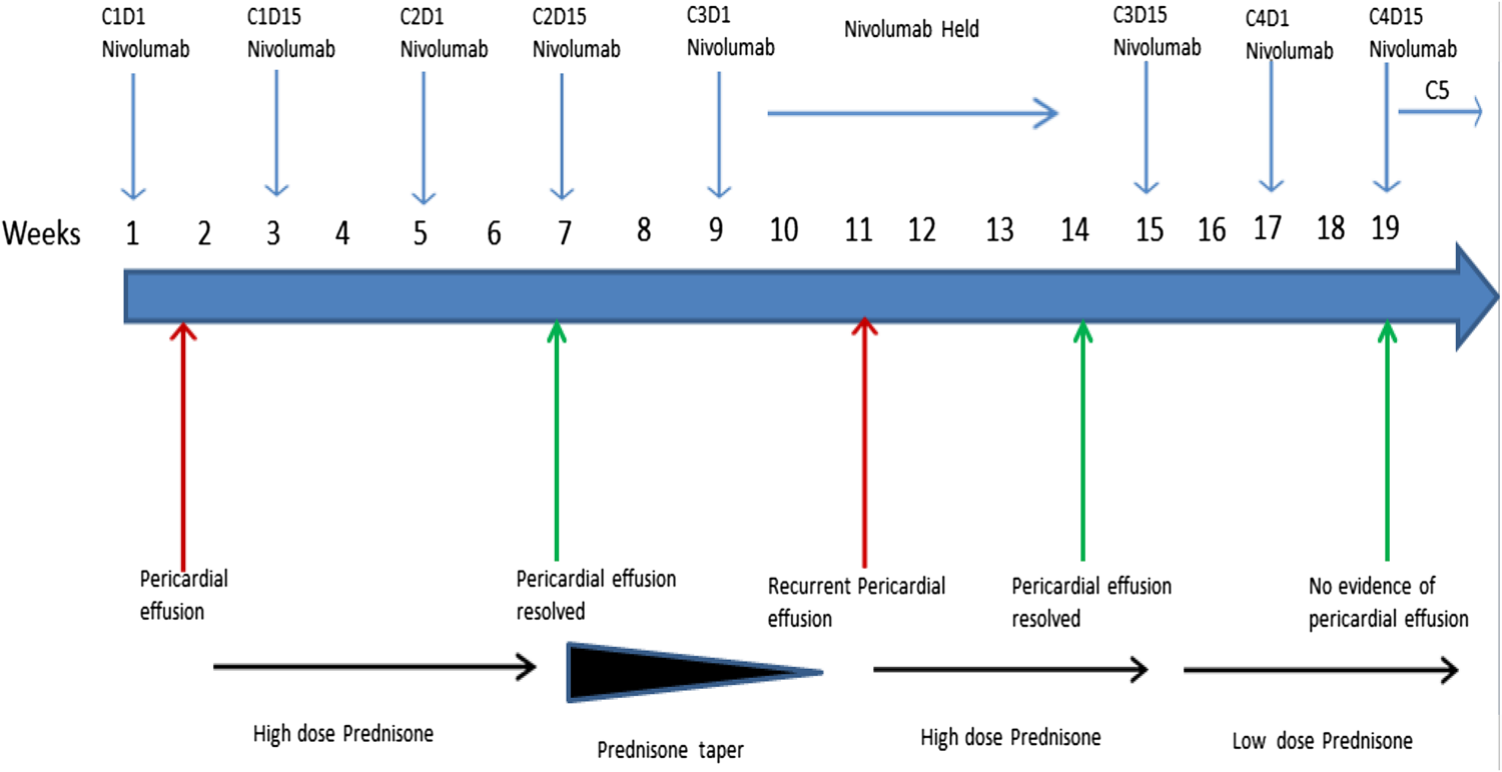
Timeline of nivolumab treatment related to the development of pericardial effusion and the use of steroid

Four days after her first dose of nivolumab, patient presented with acute shortness of breath and chest pain. Initial work up, including infectious, was unremarkable except for mildly elevated creatinine kinase of 40 mg/dl. Electrocardiogram showed sinus tachycardia with heart rate of 124 beats/min, blood pressure was 98/55 mm Hg and patient was afebrile. A CT angiography of chest showed no pulmonary embolism or pneumonitis but new-onset large pericardial effusion of 2.4 cm thickness (Fig. 1). Patient was admitted to cardiology service, transthoracic echo confirmed large pericardial effusion; posteriorly 2.1 cm, anteriorly 1.6 cm without features of tamponade. Patient was started on colchicine and fluid resuscitation for her hypotension, with symptomatic improvement. Follow-up echocardiogram showed persistent pericardial effusion. Given unclear etiology of her pericardial effusion and likely immune-related adverse event (irAE) of nivolumab, patient was started on prednisone 1 mg/kg/day. She continued to receive nivolumab treatment every 2 weeks. Follow up echocardiogram and computed tomographic scan (Fig. 2) after 4 weeks showed complete resolution of pericardial effusion and her prednisone was slowly tapered off. One week after her prednisone was discontinued, she presented again with sub-sternal chest discomfort, shortness of breath and bilateral ankle edema. Echocardiogram showed recurrence of moderate to large pericardial effusion measuring 2.8 cm posteriorly and 1.2 cm anteriorly without tamponade. She was hospitalized for observation of any hemodynamic instability. Her recurrent pericardial effusion was likely irAE from nivolumab, hence nivolumab treatment was stopped and patient was started on prednisone 1 mg/kg/day. Significant clinical improvements was noted and follow up echocardiogram after 3 weeks showed complete resolution of her pericardial effusion. Nivolumab was resumed 1 week after while maintaining patient on low dose prednisone. Patient tolerated nivolumab well and a follow up echocardiogram 4 weeks later did not show any recurrence of pericardial effusion. The timeline of nivolumab treatment related to the development of pericardial effusion and the use of steroid is depicted in Fig. 3.

## Discussion and conclusions

Immune checkpoint inhibitors have emerged as important therapeutic approach in patients with advanced NSCLC. Nivolumab is a IgG4 monoclonal antibody against PD-1 which has shown improved survival in advanced NSCLC. In two Phase III trials, nivolumab was compared to docetaxel in patients with squamous (Checkmate 017) [3] and non-squamous NSCLC (Checkmate 057) [4]. In both trials, nivolumab significantly reduced the risk of death compared to docetaxel (41 and 27% lower risk of death for squamous and non-squamous NSCLC, respectively). Nivolumab has been approved in the US and in Europe as second-line treatment for advanced NSCLC. Of note, pembrolizumab, another monoclonal antibody against PD-1, has been approved by US FDA in first-line and 2nd-line settings in patients with advanced NSCLC [5–7].

Adverse events for, immune checkpoint inhibitors differ from that of conventional cytotoxic chemotherapy. In addition to common side effects such as fatigue, immune checkpoint inhibitors have distinct immune related adverse events (irAE) [8]. These irAE are triggered by activation of autoimmune system by T cell activation. These include autoimmune colitis, hypophysitis, hypothyroidism, hepatitis, nephritis, pericarditis and pneumonitis. In clinical trial about 40% of patients experience grade 1 or grade 2 toxicity where grade 3 or 4 were reported in 10% of patients [3, 4, 9]. These irAE should be recognized and diagnosed early in order to avoid life threatening complications and are managed by administering high-dose corticosteroids. Treatment should be discontinued and patient should be supported with hormone replacement if needed and in certain cases TNF-alpha-antagonists or mycophenolate mofetil was found to be helpful [10]. Immunosuppression is a known complication of high-dose corticosteroids, but the use of it in the management of irAE in patients receiving ipilimumab, a CTLA-4 inhibitor, did not affect clinical outcomes such as survival [11].

Pneumonitis, pleural effusion and pericarditis are well-known autoimmune complications of immune checkpoint inhibitors [12, 13]. In a case series of eight patients reported by Heinzerling et al. [14], various cardiac adverse events such as myocarditis, congestive heart failure, cardiac arrest, myocardial fibrosis, cardiomyopathy, and pericarditis were reported with treatment of ipilimumab, pembrolizumab and nivolumab. The incidence of pericardial effusion during PD-1 inhibitor treatment for advanced NSCLC is rare, and only has been described in about a handful of case reports. Most of the etiology is irAE, but disease progression and infection might be the cause as well. Nesfeder and others reported a case of non-malignant pericardial effusion during nivolumab treatment for NSCLC, who was treated with pericardiocentesis and pericardial window [15]. Kushnir and Wolf reported a NSCLC patient developing massive pericardial effusion with tamponade from nivolumab, who was treated by pericardiocentesis and subsequent steroid treatment with discontinuation of nivolumab [16]. Kolla and Patel reported two cases of lung cancer with pericardial effusion and cardiac tamponade from progressive disease while receiving nivolumab; both patients received pericardiocentesis with cytological examination confirming malignancy [17]. Chu and others reported a case of cardiac tamponade from reactivation of tuberculosis during nivolumab treatment for advanced NSCLC, and successfully managed with anti-tuberculosis therapy [18].

To our knowledge, this is the first case of nivolumab-associated pericardial effusion managed successfully with conservative treatment. The initial event of pericardial effusion occurred just days after initiating nivolumab. Due to transient physiologic consequences and excellent response to high-dose corticosteroids, our patient never required invasive measures such as pericardiocentesis. Pericardial effusion recurred after steroid was tapered off while patient continued on nivolumab, indicating the pericardial effusion was irAE and unlikely malignant. Subsequently recurrent pericardial effusion responded to high-dose corticosteroids, and patient resumed nivolumab while maintaining on low-dose prednisone. The consensus from the Society for Immunotherapy of Cancer recommends discontinuing immune checkpoint inhibitor permanently in patients suffering from life-threatening consequences of irAE (grade 4), and giving corticosteroids with immune checkpoint inhibitor in patients recovered from physiologic consequences of irAE (grade 3) [19].

## Abbreviations

PD-1: programmed death 1
PD-L1: programmed death ligand 1
NSCLC: non-small cell lung cancer
irAE: immune-related adverse events
